# A synergetic combination of small and large neighborhood schemes in developing an effective procedure for solving the job shop scheduling problem

**DOI:** 10.1186/2193-1801-3-193

**Published:** 2014-04-16

**Authors:** Mehrdad Amirghasemi, Reza Zamani

**Affiliations:** SISAT, Faculty of Engineering and Information Sciences, University of Wollongong, Building 39, Wollongong, NSW 2522 Australia

**Keywords:** Job shop scheduling, Local search, Shifting bottleneck, Forward-backward mechanisms

## Abstract

This paper presents an effective procedure for solving the job shop problem. Synergistically combining small and large neighborhood schemes, the procedure consists of four components, namely (i) a construction method for generating semi-active schedules by a forward-backward mechanism, (ii) a local search for manipulating a small neighborhood structure guided by a tabu list, (iii) a feedback-based mechanism for perturbing the solutions generated, and (iv) a very large-neighborhood local search guided by a forward-backward shifting bottleneck method. The combination of shifting bottleneck mechanism and tabu list is used as a means of the manipulation of neighborhood structures, and the perturbation mechanism employed diversifies the search. A feedback mechanism, called repeat-check, detects consequent repeats and ignites a perturbation when the total number of consecutive repeats for two identical makespan values reaches a given threshold. The results of extensive computational experiments on the benchmark instances indicate that the combination of these four components is synergetic, in the sense that they collectively make the procedure fast and robust.

## Introduction

As an integrated component of computerized and flexible manufacturing systems, the Job-Shop Scheduling Problem (JSP) is encountered in many industrial contexts. The importance of this problem is two-fold. First, it has a wide-spread applicability in manufacturing, and second, despite its easy-to-state description, it is a notoriously difficult and intractable problem which provides an ideal framework to evaluate innovative algorithmic approaches. Successful approaches for this easy-to-state problem can be later modified to cope with hard-to-state scheduling circumstances.

Among many different procedures developed to cope with the JSP, those which employ local searches have the most robust and effective aspects. A local search differs from a systematic tree search in that systematic tree search expands a graph of partial solutions, whereas a local search explores a virtual graph connecting each complete solution to its neighboring complete solutions. The number of arcs in this virtual graph is affected by the neighborhood scheme employed by the local search, with the larger size of neighborhood leading to higher number of arcs and consequently larger or even impractical required computational times. That is why the endeavor of defining a proper neighborhood scheme highly determines the success of any local search algorithm.

Defining a proper neighborhood scheme for a local search is, however, involved with highly conflicting factors, in the sense that despite the fact that many neighborhood schemes seem to be only superficial variation of one another, they can easily demonstrate entirely different results. The reason of this phenomenon has been partly described by the notion of fitness landscape (Forrest and Mitchell 1993), and it seems that successful neighborhood schemes have the capability of effectively managing a trade-off between computational time and the number of arcs in their virtual graphs.

In tackling the JSP, this paper presents a procedure that combines small and large neighborhood structures. The procedure, called SLENP (Small-Large Embedded Neighborhood Search), has four synergetic characteristics of (i) making use of a forward-backward construction method for generating initial solutions, (ii) employing a small neighborhood search, (iii) using a feedback-based mechanism in generating perturbation for improving the result of the small neighborhood search, and (iv) using a large-neighborhood search for improving the overall result of the combination of the small-neighborhood search and the perturbation mechanism employed.

The feedback process employed for igniting solution perturbation is based on memorizing the values of solutions generated, and is aimed at minimizing the chance of existing any redundancy in the search. The rationale behind the use of this feedback process in performing perturbation is that any perturbation contributes to exploration but spoils any exploitation aspect of the search and hence it should be performed by extreme care. Memory undeniably is a vital constituent of any successful search, and here it has been used for igniting perturbation to avoid the same area of the virtual graph to be visited repeatedly.

Mixing different neighborhood structures is one of the building blocks of the variable neighborhood search (Hansen and Mladenović 2003), and it seems that combining small and large neighborhood schemes can have dual benefits. On the one hand, the poor decision made in a small neighborhood, which is the natural consequent of its limited scope, may be rectified, and on the other hand, because of the comparatively high quality of its initial solution, the employed large neighborhood search may require less computational effort in producing its final result.

The SLENP performs its large neighborhood search through a variant of the shifting bottleneck procedure which works both in the forwards and backward directions. By performing in the backward direction, the ordinary operations of the shifting bottleneck procedure are executed on an inverted network, called mirror network. The term mirror best reflects how, by reversing the precedence relations of the initial network, this modified network is created and why the overall solution based on this modified network can be mirrored to show a solution to the original network.

The outline of the paper is as follows. The next section starts with presenting the formulation of the job shop problem and providing a brief literature survey on the problem. Section Related works presents the related work. In Section The SLENP, the SLENP is discussed and a stepwise description is provided that describes how the procedure operates and clarifies how its different components interact with one another. Section Computational experiments presents the results of computational experiments. A summary of the results as well as the suggestions for future work are discussed in Section Concluding remarks.

## Problem formulation

The JSP consists of *n* jobs and *m* machines, with each job having a specific processing order on the machines. A typical schedule for the JSP is the allocation of jobs to the time slots of the machines to minimize the makespan. In other words, each job is comprised of a sequence of *m* operations, each to be processed on a specified machine within a particular time. The goal is to minimize the finish time of the last activity completed subject to the constraint that, once started, an operation cannot be interrupted and should continue until it has been completed. It is worth noting that makespan is a regular criterion and any method capable of handing this criterion has the potential of being modified to handle other regular criteria like total weighted flow time, weighted tardiness, weighted sum of tardy jobs, and maximum tardiness (Mati et al. 2011). The reverse is also true with the procedures handling other regular criteria, like weighted tardiness (Bülbül 2011; Kreipl 2000).

As an NP-hard problem (Lawler et al. 1982), the JSP is a notoriously difficult and an intractable combinatorial optimization problem. An evidence for its intractability is that finding the optimal solution of a relatively small problem instance presented in (Fisher and Thompson 1963), with the dimension of 10*10, despite the focus of intensive research on it, remained unsolved for 26 years until it was solved by the exact procedure developed in (Carlier and Pinson 1989). This celebrated instance, which in the literature is called ft10, is still used by many researchers to test their algorithms. The other exact procedures that have been successfully applied to small instances have been developed in (Applegate and Cook 1991) and (Brucker et al. 1994). By exploring specific knowledge about the longest path in the disjunctive graph and employing the cutting-plane method for obtaining lower bounds, these methods are aimed at applying sophisticated inference rules to cut the enumeration tree in its early expansion phase, with both of the methods able to solve the ft10 to optimality within several minutes.

**Figure 1:**
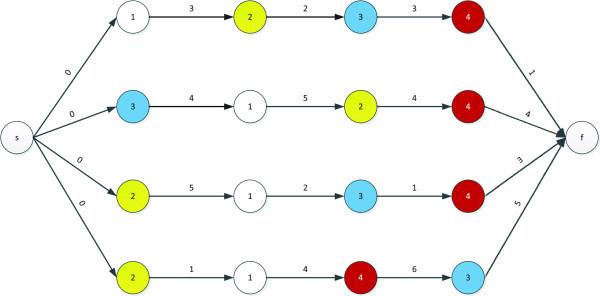
**A sample 4-machine 4-job JSP problem in which the number of the required machine has been written in each circle (operation).**

**Figure 2:**
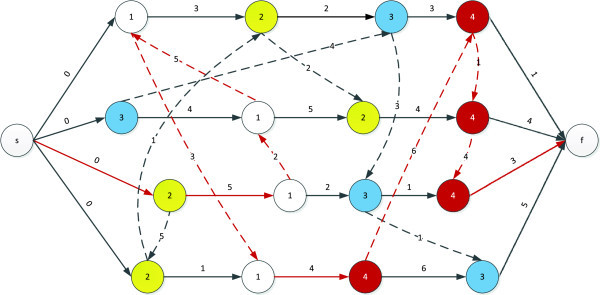
**Fixing the disjunctive arcs of the sample problem leading to the makespan of 33.**

**Figure 3:**
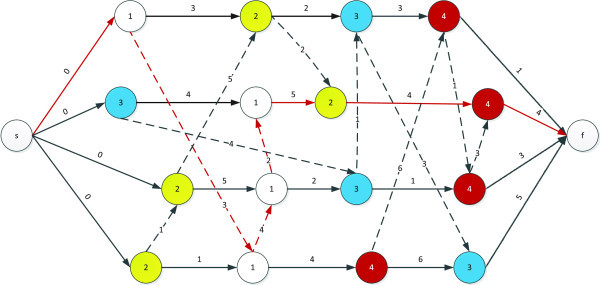
**Fixing the disjunctive arcs of the sample problem leading to the makespan of 22 (optimal solution).**

One of the most effective formulations of the JSP is performed by the use of disjunctive graphs (Brucker et al. 1994). Figure 1 shows a sample problem and Figures 2 and 3 show two different fixation settings for the disjunctive arcs of the sample problem. As is seen, the makespan of the problem, as the longest path from the starting to the ending node, is different for Figures 2 and 3. In effect, Figure 3 shows an optimal fixation setting for the disjunctive arcs, which has led to the optimum makespan of 22.

**Figure 4:**
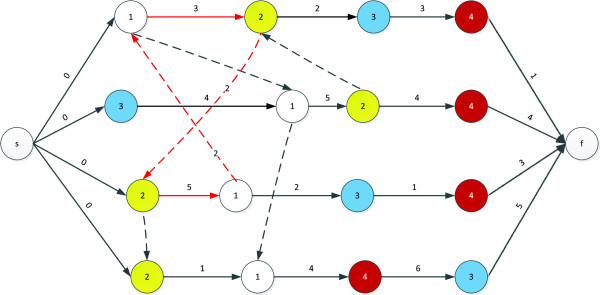
**An infeasible fixation of disjunctive arcs leading to a loop of 1-2-9-10-1.**

Since all operations executed on the same machine require a given order, the notion of feasible order plays a key role in such graphs, with machine *i* being associated with the order π_i_, which shows the permutation of jobs on that machine. An order, Π, which consists of {π_1_, π_2_,....,π_m_} is feasible if it does not introduce any loop in the graph. Figure 4 shows an infeasible fixation of disjunctive arcs and the resultant loop produced.

Denoting the completion time of the last completed job with *makespan*, the JSP can be simply formulated as follows:1

## Related works

Solution strategies presented for the JSP range from artificial bee colony optimization and hybrid genetic tabu searches (Banharnsakun et al. 2012; Meeran and Morshed 2012; Zhang et al. 2013; Zhang et al. 2008b) through dynamic and linear programming (Gromicho et al. 2012; Bülbül and Kaminsky 2013) to path relinking and particle swarm optimization (Pongchairerks 2014; Nasiri and Kianfar 2012). In an overview of scheduling models presented in (Framinan et al. 2014) several of these strategies have been examined. Non-exact solution strategies for the JSP, to which the method presented in this paper belongs, can be categorized into six different categories, namely (i) construction methods, (ii) local searches, (iii) metaheuristics, (iv) evolutionary algorithms, and (iv) hybrids. Interestingly, nearly all successful techniques in these categories model the JSP as a disjunctive graph. In each category, only those works have been discussed which have affected the SLENP.

Construction methods build a solution progressively, starting with a null schedule and expanding it gradually until a full schedule is obtained. In the process of creating a full schedule, a sequence of intermediate partial schedules is created, with each partial schedule expanding the previous partial schedule. In general, myopic decisions are the backbone of such methods in the progressive expansion of the intermediate partial schedules. Priority-based techniques are the oldest techniques classified as construction methods.

The method presented in (Giffler and Thompson 1960) is one of the most effective methods in the category of construction methods. This method is able to accept a set of priorities and create either an active or a non-delay schedule through a dispatching mechanism which schedules eligible operations one at a time based on their priorities.

The second major construction method is the shifting bottleneck procedure (SBP) (Adams et al. 1988), which decomposes the JSP into several, *m*, one machine problems and solves each problem to optimality with the Carlier’s method. As an intricate algorithm, the SBP repeatedly redirects the search towards scheduling the machine which imposes the most severe constraint in the sense of increasing the objective function.

As the counterpart of gradient optimization in continuous spaces, local searches probe discrete spaces through the fundamental notion of *move* to find local optimal solutions. In effect, modern local searches are now the leading procedures in solving the JSP, and this is mainly due to effective neighborhood schemes developed in the last three decades. In general, local searches convert a complete solution to another complete solution through local changes.

A major point with local searches is that they cannot be effective unless they exploit the structure of the problem through a proper definition of a neighborhood structure and an effective mechanism for the manipulation of such a structure. Nearly all effective neighborhood structures for the JSP are based on the basic notion of critical path in a resolved disjunctive graph. In effect, each critical path represents the longest route through operations and its length is equal to the makespan. Major neighborhood structures for the JSP are as follows.

N1 neighborhood has been proposed in (Van Laarhoven et al. 1992) and defines a move by interchanging two successive operations of the same machine on a critical path. The design of N1 has been made based on two principles: (i) changing the order of two non-critical operations cannot improve the solution and may only create a cycle in the disjunctive graph, and (ii) changing the order of two adjacent operations cannot create a cycle.

N2 neighborhood (Dell'Amico and Trubian 1993) can reverse more than one arc on the critical path. Assuming *i* and *j* are two consecutive operations of the same block and one of them is at an extreme point of the block, the predecessor of *i* and the successor of *j* can also be subject to reversal with their predecessors and successors, respectively.

In the N3 neighborhood (Dell'Amico and Trubian 1993), a sequence of three operations on the critical path can be reversed subject to the condition that such a reversal does not lead to any loop. As an extension of N1, N3 is not limited to the reversal of triplets and includes the interchange of a pair of operations as well.

In N4 neighborhood (Dell'Amico and Trubian 1993), each operation of a block can move to any location of the block, subject to creating no cycle. Unlike in the other three neighborhood schemes, which are based on adjacent interchanges of operations, in this neighborhood a shift is performed. Based on this shift, an operation jumps over several other operations in its corresponding block to the left or right. In effect, this neighborhood can be considered as an expansion of all other previous ones.

As a restricted version of both N1 and N4, N5 is a neighborhood scheme, developed in (Nowicki and Smutnicki 1996), in which the first two or the last two operations of each block are interchanged. The only exceptions are the first and last blocks, in which only their last and first two operations are interchanged, respectively.

The rationale behind the development of N5 is that the size of N1 is large and includes a large percentage of moves that cannot lead to any improvement. These unfruitful moves are those which are involved with two internal operations in the corresponding block. The removal of these unfruitful moves out of N1 leads to the creation of N5, which includes a restricted collection of highly effective moves.

However, despite using such highly effective moves, a drawback with N5 neighborhood is that its corresponding search space is disconnected. This disconnection removes any guarantee for the existence of a path between an optimal solution and an arbitrary seed. In comparison with N4, N5 is involved with the reversal of only one disjunctive arc, and this makes its corresponding neighborhood considerably smaller.

In N6 neighborhood (Balas and Vazacopoulos 1998), each operation of a block can move precisely after the last or before the first operation of the black, subject to creating no cycle. N6 is very close to N4, and the major difference existing between these two neighborhood schemes is that N4 allows each operation of the block to move to any other location of the block, subject to creating no cycle. This makes the size of N6 slightly smaller than that of N4. N6 has been extended to N6’ in (Zhang et al. 2008a) by being allowed to move the first or the last operation of the block into the interior operation inside the block.

It is worth mentioning that in the literature these notations are not unique and different authors have used different notations. For instance, whereas in (Blazewicz et al. 1996), N5 is referred to the same neighborhood we mentioned, in (Vaessens et al. 1996) N5 is used to refer to the neighborhood scheme developed in (Adams et al. 1988), which, as a very large neighborhood, can completely change the order of operations on one machine. That is why in this paper, we refer to that neighborhood schemes as N5’.

Local searches, in general, and neighborhood schemes, in particular, are mainly used in the context of metaheuhurtics. Tabu searches, as part of metaheuristic category, select improving moves, and are aimed at avoiding to return to the solutions they have visited recently. In the cases where no improving move exists, simply the least disapproving move is chosen. The tabu search presented in (Nowicki and Smutnicki 1996) has been one of the most effective searches presented for the JSP. Although it owes its effectiveness to both the employed neighborhood structure and the balance it maintains between diversification and intensification, restarting the search form elite solutions plays a crucial role in its success. In this metaheuristic, the search is controlled through a backtracking scheme, and the seed are provided through generating active schedules. The backtracking process embedded in the search causes the search to restart from various high quality solutions encountered from the beginning of the search. In other words, it recovers elite solutions with which to restart the search.

In the JSP whose fitness landscape has big valley structure (Pardalos et al. 2010), the recovery of elite solutions and then restating the search with these solutions using different parameters has proved to be very effective. Because of a random setting, even with using the same high quality solution as a seed, each restarting initiates finding a new promising trajectory. It is through these trajectories that the big valley structure of the JSP is exploited and high quality solutions are obtained.

As two other effective tabu searches, we can name those presented in (Taillard 1994) and (Barnes and Chambers 1995), with both using N1 as their neighborhood structure. Whereas the first procedure changes the size of tabu iteratively and calculates the objective function value of each neighbor approximately, the other procedure has a fixed-length tabu list and computes the objective function value of each neighbor exactly. For generating a seed, several non-delay schedules are generated and the best one is selected.

As another metaheurstic, the guided local search presented in (Balas and Vazacopoulos 1994), uses a variable neighborhood search in escaping local optimality. The main difference between this search and a typical variable neighborhood search is that it uses a tree whose nodes correspond to the orientations, with each descendant node being a neighbor of its parent node. In (Mattfeld 1996), this search has cleverly been classified as a variable depth search due to the famous algorithm of (Lin and Kernighan 1973), which has first been applied to the Travelling Salesman Problem (TSP). As another variable neighborhood search for the JSP we can mention the VNS presented in (Wang and Zhang 2011).

The variable depth search starts with an initial solution, and in each iteration of the search, it starts with the best solution found in the previous iteration. Unlike in tabu search, each iteration performs not one but a number of potential profitable moves, which in general may be worsening moves, and during the corresponding iteration never reverses any of those moves.

Despite the fact that in the next iteration, previously prohibited moves are allowed to be performed again, the list of forbidden moves in the variable depth search grows much faster than that in tabu search. The other variable depth search procedure developed for the JSP, is the procedure presented in (Dorndorf and Pesch 1993). Unlike in the TSP, this search has not been extremely successful for the JSP. It seems that this is partly due to the complicated structure of the JSP, which makes potentially profitable moves unrecognizable.

In (Lourenco 1995; Lourenço and Adviser-Shmoys 1993), a combination of N1 and a large step optimization search has been employed to tackle the problem. In the corresponding large step optimization, randomly two machines are selected and all disjunctive arcs related to these two machines are removed. Then, a new order is found for each of these machines through using the Carlier’s method (Carlier 1982), which solves one machine problem to optimality.

The fourth category, genetic algorithms, is not much related to our work, and the most successful genetic algorithms provided for the JSP include those presented in (Yamada and Nakano 1992), (Dorndorf and Pesch 1995), (Falkenauer and Bouffouix 1991), and (Gonçalves et al. 2005). Finally, we briefly survey hybrids as the last category. They comprise a variety of algorithms ranging from genetic (Qing-dao-er-ji and Wang 2012) through ant bee-based (Zhang et al. 2013) to differential evolution hybrids (Ponsich and Coello Coello 2013).

The first related hybrid discussed is the algorithm based on global equilibrium presented in (Pardalos and Shylo 2006). This algorithm, which is called Global Equilibrium Search (GES), has some common features with simulated annealing algorithm. In each stage of the search, GES collects information about the solution space for its next stages, and similar to the SA, the GES performs the search as a chain of temperature rounds.

The procedure also employs a local search which uses two neighborhood structures. The first neighborhood structure is N1 and the second structure manages to move each operation on the block either to the beginning or to the end of its corresponding block, similar to N4. The authors have modified an accelerating method in literature for the evaluation of moves in their second neighborhood structure. This method, which instead of computing the exact value associated with a move, calculates its tight lower bound at the cost of negligible computational effort, has significantly contributed to the effectiveness of the procedure.

The next related work in the hybrids is a filter-and-fan approach presented in (Rego and Duarte 2009). The authors have presented a filter and fan approach for solving the JSP. The SBP (Shifting bottleneck procedure) has been used both for generating initial solutions and enhancing the final solutions as a post optimization procedure. It incorporates a tree search for restricting the solution space and works similar to the beam search.

Whereas beam search works in construction heuristics, filter and fan search, as its natural generalization, can work both in construction heuristics and local searches. When used in local searches, it places a local optimal solution at the root of the search tree, and the best *m* solutions obtained in this process are located at level 1, among the neighbors of these *m* solutions, the best *m* neighbors are selected and are placed in level 2. In effect, each level consists of *m* nodes that are selected among the best neighbors of nodes existing in its previous level. By using a hash mechanism, any repeat in this search tree is prevented. After generating *k* solutions and selecting the best solution obtained in the process, it becomes the root of the tree, and the search restarts.

The employed filter and fan procedure can work based on the first-improvement strategy as well. This means that whenever in the process, the root is improved, the enhanced solution becomes the root, and the best *m* solutions obtained in the previous search tree are placed in the first level of the next search tree.

The next related work in hybrids is a TS/SA algorithm presented in (Zhang et al. 2008a). The procedure developed by these authors is based on their conjecture that the quality of solutions obtained by the tabu search is determined based on the quality of the initial solutions. That is why they use a simulated annealing algorithm to generate high quality initial solutions for a tabu search. In other words, the main principle guiding this search is that SA generates elite solutions and TS improves the solutions generated by SA.

These kinds of integration are usually very effective. For instance, in (Huang and Liao 2008) ant systems are combined with tabu searches and provide assistance for a decomposition method inspired by the shifting bottleneck procedure, used as the construction method of the procedure. Having briefly reviewed the related work, we can now describe the SLENP.

## The SLENP

The SLENP combines small and large neighborhood schemes in coping with the job shop problem. Four modules have been combined to create this procedure. The first module is aimed at generating semi-active schedules by a forward-backward method called Semi-Active Schedule Generator (Forward-Backward-SASG). The second module is based on a local search that manipulates a small neighborhood structure guided by a tabu list, and the third module includes a feedback-based mechanism for perturbing the solutions generated. A very large-neighborhood local search, which is guided by a forward-backward shifting bottleneck (Forward-Backward-SBP) method, comprises the fourth module. Whereas the shifting bottleneck mechanism and tabu list are used as a machinery to manipulate the neighborhood structures, the perturbation mechanism diversifies the examined solutions, and the semi-active generator generates initial schedules.

The employed small neighborhood structure is highly restrictive, and the reason we have used semi-active, instead of active schedules, is that in (Jain et al. 2000), through computational experiments, it has been shown that semi active schedules better match with restrictive neighborhood structures. The conclusion made in (Jain et al. 2000) is partly based on semi-active schedules employed in (Nowicki and Smutnicki 1996). It should be noticed that when the employed neighborhood structure is not restrictive, active schedules perform better than semi-active schedules. The reason is twofold. First, makespan is a regular criterion and the optimum belongs to the set of active schedules. Second, active schedules are a subset of semi-active schedules, and, on average, are of higher quality than semi-active schedules.

In the procedure, the repeats are detected by a feedback mechanism called repeat-check, which causes perturbation to occur whenever the total number of successive repeats for two identical values of the makespan reaches a given threshold. The shifting bottleneck module manipulates a large-neighborhood and is aimed at enhancing the solution obtained by the other three modules. To improve the results obtained by the shifting bottleneck method, a forward/backward mechanism has been added to it.

**Figure 5:**
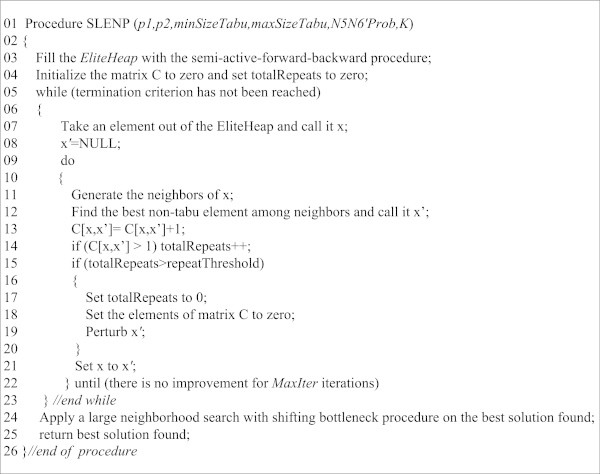
**The c-type pseudocode of the SLENP.**

The pseudocode of the SLENP has been represented in Figure 5. The integrating part of the pseudocode is *Elite Heap,* which is a priority queue for keeping high quality solutions for possible improvement and releasing them based on their quality. First, at line 3, the *EliteHeap* is initially filled by repeatedly calling the Forward-BackwardSASG procedure. This is performed through generating *n* solutions by the Forward-BackwardSASG procedure, and selecting the best *m* solutions among them. The Forward-BackwardSASG procedure has its own pseudocode and will be discussed in detail.

After the filling of the *EliteHeap*, the main loop in pseudocode starts at line 5. The main goal of this loop is to make possible improvement in the solutions located in the *EliteHeap*. Line 7 removes a solution from the *EliteHeap* and line 9 performs a limited tabu-search on this solution. In this tabu search, the tabu list includes elements which show the sequence of operations on a particular machine. In this tabu search, both N5 and N6′ neighborhoods are used. As discussed, N6′ is a version of N6 in which the possibility of moving the starting and ending operations of the block to the interior positions of the block have been considered.

The employed tabu list is aimed at determining whether, within a particular short-term period, a potential solution has been visited and decreases the possibility of repeatedly visiting the same sequence of solutions. Since this particular short-term memory cannot exclude the large sequences of repeats, lines 13 and 14 record the occurrence of every two consecutive solution values, and line 15 prevents any such possible repeat through making a perturbation in the current solution, aiming at further decrease in the possibility of repeats.

The performance of such perturbation depends on detecting a large sequence of repeats. For this purpose, if *x* has been followed by *x*’ previously, C[*x*,*x*’] > 1, the value of *totalRepeats* is incremented at line 14, and as soon as *totalRepeats* exceeds a certain threshold, the current solution is perturbed using the N1 neighborhood. As lines 17 and 18 indicate, with each perturbation, the history of the recorded solution values is discarded. The reason for discarding this history is that any perturbation changes the course of possible repeats. Line 22 ensures that the procedure is repeated until there is no improvement for *MaxIter* iterations. The loop terminates at line 23.

**Figure 6:**
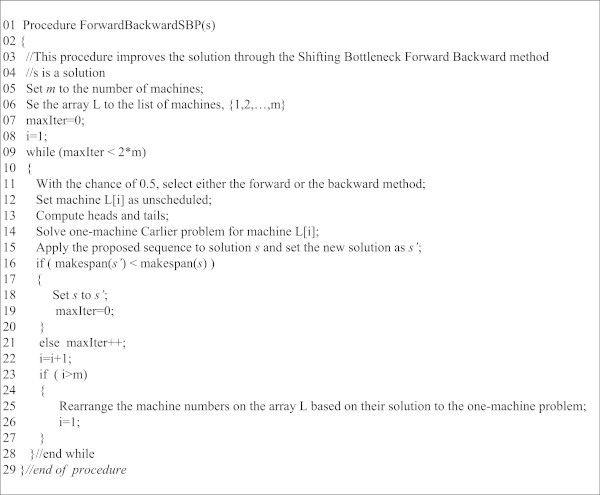
**The c-type pseudocode of the ForwardBackwardSBP module.**

The best solution obtained in the loop undergoes an iterated forward-backward, one-machine post-optimization process for possible further improvement. The loop terminates at line 23, and line 24 applies a large-neighborhood search to the best solution obtained. This is performed by calling *ForwardBackwardSBP*, whose complete pseudocode has been given in Figure 6.

This complete pseudocode describes a modified version of the post-optimization phase of the shifting bottleneck procedure (SBP) (Adams et al. 1988). The modification performed includes adding a backward process to the procedure. The pseudocode starts with initializing a list of machines, L*,* at line 5.Then a loop starts at line 9. In each iteration of this loop, at line 12 the status of a machine is set to *open* (i.e. “not scheduled”), and at line 14 the sub-problem corresponding to that machine is given to the Carlier procedure to be solved.

Based on line 11, the procedure randomly switches from the forward case to the backward case and vice versa. After solving the corresponding one machine problem with the Carlier method at line 14, the output sequence is applied to the solution and the makespan is calculated at line 15. Then, line 16 replaces the previous sequence with the new one if the new sequence is of higher quality than the previous one. Lines 7, 9, 19, 21 ensure that the procedure is terminated if there is no improvement for 2*m* consecutive iterations. As lines 23 through 27 show, after each full optimization cycle for all *m* machines, the machine numbers in the list *L* are rearranged based on their solution cost to the one-machine problem, with the machines producing the longest makespan sitting in the top of the list and those producing the smallest makespan sitting in the bottom of the list. The pseudocode terminates at line 29.

**Figure 7:**
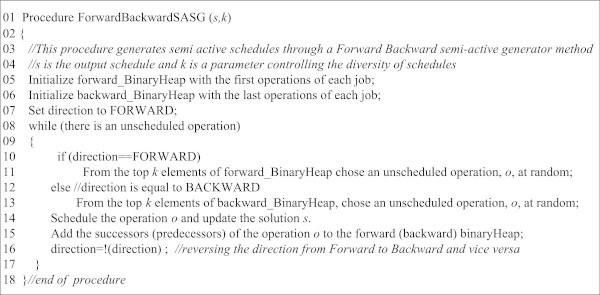
**The c-type pseudocode of the ForwardBackwardSASG.**

As mentioned, the *EliteHeap* is filled by repeatedly calling the Forward-BackwardSASG procedure. The pseudocode of this procedure has been presented in Figure 7. The pseudocode, which generates semi-active schedules, starts with lines 4 and 5 and initializes a forward and backward binary heap, respectively. Then with a loop starting at line 8, forward and backward processes are used alternatively, starting with the forward process, which is set at line 7. Depending on whether the forward or backward process is in place, an element is taken from the forward or backward heap in lines 11 and 13, respectively.

The pseudocode constructs a solution by iteratively sequencing jobs on machines. This is done in the two directions of forward and backward alternatively, in the sense that in each iteration based on the value of the *direction* variable, an operation is scheduled on the beginning or the end of the schedule, respectively. Since in lines 11 and 13 from the top *k* elements of forward_BinaryHeap or backward_BinaryHeap, an element is chosen, and larger values of *k* can cause further diversification, the parameter *k* plays a key role in the quality of solutions generated.

The smaller values of *k* lead to generating a limited number of solutions, all in the same high quality region. On the other hand, when the value of *k* is increased, say to 3 or 5, the diversification is increased at the cost of decreasing the quality of regions. Since for filling the *EliteHeap*, this module is called for *n* times and among the *n* solutions generated the best *m* solutions are selected, the values of each of the three parameters *k, m*, and *n* should be selected based on the other two parameters.

The other issue contributing to the effectiveness of the procedure is the forward-backward mechanism embedded in the Carlier’s method as the key component of the shifting bottleneck procedure. Both in the forward and backward processes, it is vital that the sub-solution provided by the Carlier’s procedure matches with the current solution, in the sense that the Carlier’ procedure should not introduce any loop in the current disjunctive graph. As it has been stated in (Adams et al. 1988), introducing loops through the Carlier’s procedure is not a common occasion and rarely can happen in practice. In our implementation, both in the forward and backward processes, we have prevented loops as follows.

Suppose, that the Carlier’s procedure suggests 71, 78, 30, 1, 42, and 35 as the sequence of operations that should be processed, one after another, on the corresponding machine. Now in the disjunctive graph, we find all successors of the operation 35 and make sure that none of the operations before the operation 35 in the proposed sequence by the Carlier’s procedure is among these successors. Then we find all successors of the operation 42, and make sure that none of the operations before the operation 42 is among them. Checking for the existence of any violation continues until we find that the operation 71 is not among the successors of the operation 78.

In case of encountering any violations, they are recorded, and the Carlier’s procedure is called again, albeit with the set of recorded violations as a constraint for being avoided. This process is repeated until the sub-solution provided by the Carlier’s procedure matches with the current solution. The employed tabu search selects improving moves and avoids returning to the solutions it has visited recently.

## Computational experiments

The SLENP has been implemented in C++ and compiled via GNU GCC compiler on a DELL PC with 2.2 Ghz speed. The benchmark problems to which the procedure has been applied include 43 instances extracted from ORLIB site managed by Brunel University, UK. The selected instances comprise a combination of representative problems collected from the literature. They range from 6 × 6 to 20 × 20 in size, with the first number showing the number of jobs and the second number showing the number of machines. They include 3 instances, *ftxx*, from (Fisher and Thompson 1963), 11 instances, *laxx*, from (Lawrence 1984), 5 instances, *abzx*, from (Adams et al. 1988), 10 instances, *orbxx*, from (Applegate and Cook 1991), 4 instances, *ynx*, from (Yamada and Nakano 1992), and 10 instances, *swvxx*, from (Storer et al. 1992).

The procedure has 11 parameters, and most of these parameters have been set in terms of the number of jobs, *n*, and the number of machines, *m*. In setting the parameters, care has been taken to increase the exploration power of the procedure with respect to the increase in *m*, and ⌊*n*/*m*⌋. Table 1 shows how these parameters have been set. A brief description of these parameters is as follows; (i) *TotSol* denotes the number of total initial solutions generated, (ii) *k* represents diversification parameter of the Forward-BackwardSASG module which controls the diversity of initial solutions, (iii) *EliteHeapSize* indicates the size of EliteHeap, (iv) *PerturbProb* represents the chance by which the solution is perturbed after being removed from eliteheap, (v) *MaxIterNonImprov* denotes the number of iterations after which the tabu search stops if no improvement occurs in makespan, (vi-vii) *TabuSize*_*min*_ and *TabuSize*_*max*_ show the extremes of the bound in which the size of the tabu list as a uniform random variable can change in each run, (viii-ix) *RepeatTolerance*_*min*_, and *RepeatTolerance*_*max*_ represent the extremes of the bound in which the tolerance for accepting consecutive makespan repeats is changed randomly, (x) *N5N6′Prob* denotes the chance of selecting N5 neighborhood in each iteration and consequently the chance of selecting N6′ neighborhood is *1-N5N6′Prob*, (xi) *TripleMovesProb* represents the probability by which at the start and the end of the critical block a triple move is performed.

**Table 1:** The value of each parameter as either a constant or a function of the number of jobs (n) and the number of machines (m)

Parameter	Value
*TotSol*	*n.m*+1000
*k*	10
*EliteHeapSize*	500
*PerturbProb*	
*MaxIterNonImprov*	
*TabuSize*_*min*_	
*TabuSize*_*max*_	
*RepeatTolerance*_*min*_	
*RepeatTolerance*_*max*_	
*N5N6’Prob*	0.1
*TripleMovesProb*	0.6

As stated in the previous section, the SLENP uses both N5 and N6′ as its small neighborhood. That is why among the parameters described above, the parameter *N5N6′Prob* has been used to determine the chance of selecting each of the two neighborhoods. Using one of the two neighborhoods randomly causes that starting with the same initial starting point leads to different courses of actions and consequently to different solutions, improving the diversity of solutions generated and increasing the chance of escaping local optimality.

**Figure 8:**
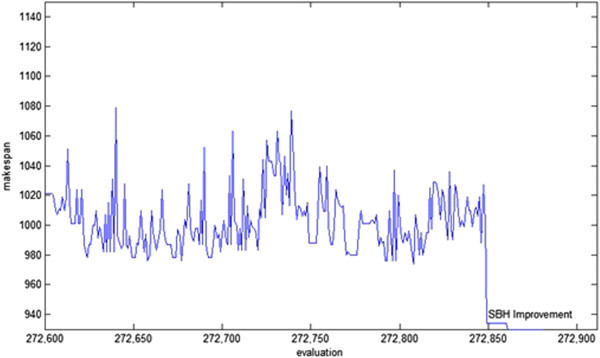
**Solution value (makespan) per each schedule evaluation for the instance ft10.**

Before presenting the performance of the procedure for the benchmark instances and comparing the obtained results with the best available solutions, we first show the results of its operations on the *ft10*, which is the most famous instance. Figure 8 shows the solution value (makespan) per each schedule evaluation. As is seen, the makespan has converged towards 930, which is the optimal solution of the problem. It is worth mentioning that value of 930 has been found after applying the shifting bottleneck improvement heuristic in the final stage of the procedure. The shifting bottleneck improvement heuristic has been executed after the procedure has evaluated 272850 schedules, and has been performed on a solution whose makespan was 934.

**Figure 9:**
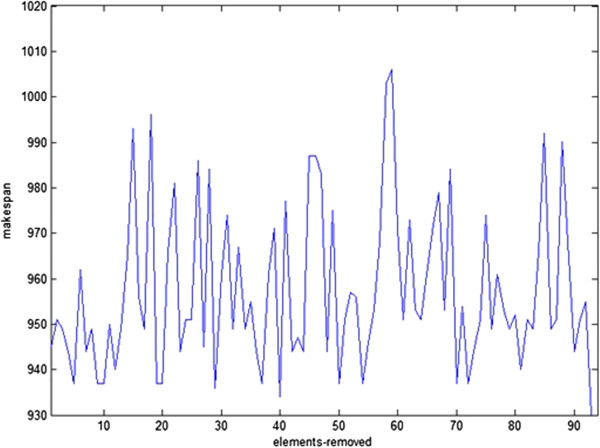
**Best makespan achieved per each element removal from the elite heap.**

Figure 9 shows the best makespan obtained for each element removed from the elite heap. In effect, for 90 different elements taken from the elite heap, this figure has presented the trend in which the corresponding element has been improved in the search. The 90 different peaks in the figure correspond to the 90 solutions taken out from the heap and each shows the makespan of the corresponding element. Associated with each peak is a dip which shows the makespan of the best solution obtained.

**Figure 10:**
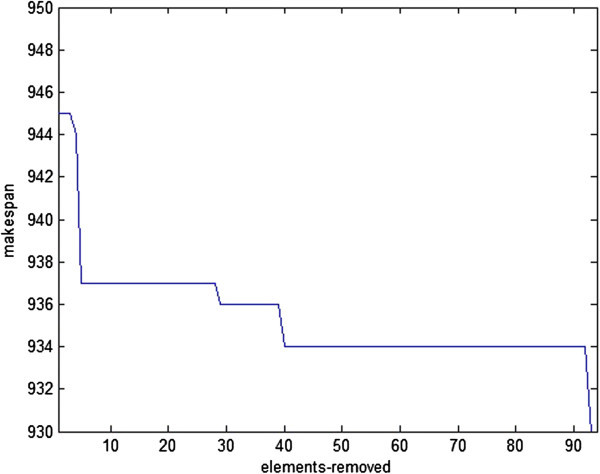
**The changes of global best solution per each removal from the elite heap.**

As is seen in Figure 9, each of the elements taken from the heap has led to a different solution, and sometimes high quality elements taken from the heap have produced solutions which cannot compete with solutions produced by low quality elements. The trend, however, is towards the improvement of the final solution. Figure 10 shows such a trend by depicting the changes in the global best solution per the removal of each element from the elite heap.

Now we present the results of applying the procedure to the entire benchmark instances selected. To remove the effect of the random seed, in line with other procedures, for each instance, the SLENP has been run for 10 times each with a different random seed. The time allowed for each run is *n*(9*n*-60)/*m* seconds for instances with *n* ≥ 10 (jobs) and 1 second for the instances with *n* < 10. Moreover, since in the case of availability of the optimal makespan it is given to the procedure as an input, the procedure can stop as soon as a solution with such quality is achieved.

With respect to performance, Table 2 compares the procedure with one of the fastest available procedures for the JSP, namely TSSA (Tabu Search Simulating Annealing) (Zhang et al. 2008a). In this table, %DEV_best_ represents the deviation percentage of the obtained solution from the best available solution in the literature, BKS, and has been obtained based on the formula of (*s*-*BKS*)/*BKS*, with *s* being defined as the best solution returned by procedure. The running times of the TSSA have been reported on a Pentium IV 3.0 Ghz CPU.

**Table 2:** **Comparing the performance of the SLENP with that of TSSA**

				SLENP					TSSA			
**Instance**	**Size**	**LB**	**BKS**	**Best**	**%DEV**_best_	**T**_best_	**Avg**	**T**_avg_(s)	**Best**	**%DEV**_best_	**Avg**	**T**_avg_
ft06	6 × 6	55	55	**55**	0.000	0	55	0.005	–	–	–	–
ft10	10 × 10	930	930	**930**	0.000	0.455	930	9.224	930	0.000	930	3.8
ft20	20 × 5	1165	1165	**1165**	0.000	0.48	1165	2.727	–	–	–	–
la19	10 × 10	842	842	**842**	0.000	0.13	842	0.776	842	0.000	842	0.5
la21	15 × 10	1046	1046	**1046**	0.000	5.3	1046.7	15.216	1046	0.000	1046	15.2
la24	15 × 10	935	935	**935**	0.000	10.29	936.5	20.360	935	0.000	936.2	19.8
la25	20 × 10	977	977	**977**	0.000	6.2	977	13.699	977	0.000	977.1	13.8
la27	20 × 10	1235	1235	**1235**	0.000	9.08	1235	31.980	1235	0.000	1235	11.7
la29	20 × 10	1152	1152	1162	0.868	86.64	1163.5	40.024	1153	0.087	1159.2	63.9
la36	15 × 15	1268	1268	**1268**	0.000	3.4	1268.3	12.937	1268	0.000	1268	9.9
la37	15 × 15	1397	1397	**1397**	0.000	1.97	1397	9.181	1397	0.000	1402.5	42.1
la38	15 × 15	1196	1196	**1196**	0.000	4.35	1198.6	14.836	1196	0.000	1199.6	47.8
la39	15 × 15	1233	1233	**1233**	0.000	1.31	1233.6	19.099	1233	0.000	1233.8	28.6
la40	15 × 15	1222	1222	1224	0.164	6.57	1226.6	15.926	1224	0.164	1224.5	52.1
abz5	10 × 10	1234	1234	**1234**	0.000	0.58	1234	3.545	–	–	–	–
abz6	10 × 10	943	943	**943**	0.000	0.12	943	0.151	–	–	–	–
abz7	20 × 15	656	656	662	0.915	39.04	664	64.822	658	0.305	661.8	85.9
abz8	20 × 15	645	665	668	0.451	106.52	672.4	55.935	667	0.301	670.3	90.7
abz9	20 × 15	661	678	688	1.475	98.7	689.5	35.820	678	0.000	684.8	90.2
orb01	10×10	1059	1059	**1059**	0.000	0.7	1059.6	5.961	1059	0.000	1059	3.5
orb02	10×10	888	888	**888**	0.000	0.14	888	0.475	888	0.000	888.1	6.4
orb03	10×10	1005	1005	**1005**	0.000	0.365	1005	7.415	1005	0.000	1012.5	13.8
orb04	10×10	1005	1005	**1005**	0.000	0.155	1006.2	7.580	1005	0.000	1008.3	14.3
orb05	10×10	887	887	**887**	0.000	1.23	887	12.093	887	0.000	888.6	6.6
orb06	10×10	1010	1010	**1010**	0.000	0.23	1010.9	9.165	1010	0.000	1010	8.5
orb07	10×10	397	397	**397**	0.000	0.14	397	0.284	397	0.000	397	0.5
orb08	10×10	899	899	**899**	0.000	2.26	899	6.020	899	0.000	902.5	7.2
orb09	10×10	934	934	**934**	0.000	0.18	934	0.509	934	0.000	934	0.4
orb10	10×10	944	944	**944**	0.000	0.15	944	0.176	944	0.000	944	0.3
yn1	20×20	826	884	892	0.905	66.63	897.7	40.040	884	0.000	891.3	106.3
yn2	20×20	861	907	911	0.441	1.78	913.4	62.312	907	0.000	911.2	110.4
yn3	20×20	827	892	900	0.897	59.85	903.1	42.178	892	0.000	895.5	110.8
yn4	20×20	918	968	982	1.446	49.81	986.8	56.047	969	0.103	972.6	108.7
swv01	20×10	1407	1407	1437	2.132	115.33	1458.5	60.642	1412	0.355	1423.7	142.1
swv02	20×10	1475	1475	1505	2.034	92.27	1520	64.431	1475	0.000	1480.3	119.7
swv03	20×10	1369	1398	1426	2.003	82.68	1434	48.665	1398	0.000	1417.5	139.1
swv04	20×10	1450	1470	1511	2.789	66.47	1517.8	57.664	1470	0.000	1483.7	143.9
swv05	20×10	1424	1424	1475	3.581	45.461	1492.3	58.092	1425	0.070	1443.8	146.7
swv06	20×15	1591	1678	1730	3.099	43.94	1738.2	81.892	1679	0.060	1700.1	192.5
swv07	20×15	1446	1600	1632	2.000	88.79	1648	68.546	1603	0.188	1631.3	190.2
swv08	20×15	1640	1756	1807	2.904	147.39	1814.1	71.765	1756	0.000	1786.9	190
swv09	20×15	1604	1661	1701	2.408	126.12	1707.5	68.661	1661	0.000	1689.2	193.8
swv10	20×15	1631	1754	1812	3.307	122.66	1820.6	85.666	1754	0.000	1783.7	184.6

Table 2 shows that, in 25 out of 43 cases, the SLENP has been able to find the best known solution for the corresponding benchmark instance. As is seen in Table 2, for four of the benchmark instances, the TSSA has no corresponding output. Removing these four rows out of consideration, the following conclusions can be drawn. In 53.8%, 21/39, of cases, the SLENP has generated solutions with the same quality as those generated by the TSSA and in general the solutions generated by the TSSA are on average around 1.05% better than those generated by the SLENP. However, the solutions produced by the SLENP have been obtained on average 112.85% faster than those generated by the TSSA. Since both procedures have used only a single processor, taking the difference between the clocks pulses on which these two procedures have been run, 2.2 versus 3.0 Ghz, implies the chance that this speed percentage may be larger than the value presented.

Also since in (Chassaing et al. 2014), a comparison has been made among several procedures based on their performance on solving la01 to la40 instances, we have tested our procedure on these instances as well. It is worth noting that despite the fact some of these instances were included in our first experiments, we have solved them again, with new initial random solutions. The results have been shown in Table 3. As is seen, except for three instances, the SLENP has been able to find the optimal solutions of all instances. One of these instances belongs to what those authors have classified as strongly large instances, and the other two instances belong to what they have classified as large instances.

**Table 3:** **The performance result on la01 to la40**

			SLENP				
**Instance**	**Size**	**BKS**	**Best**	**%DEV**_best_	**T**_best_(s)	**Avg**	**T**_avg_(s)
la01	10 × 5	666	666	0.000	0.00	666	0.00
la02	10 × 5	655	655	0.000	0.06	655	0.07
la03	10 × 5	597	597	0.000	0.06	597	0.09
la04	10 × 5	590	590	0.000	0.05	590	0.06
la05	10 × 5	593	593	0.000	0.00	593	0.00
la06	15 × 5	926	926	0.000	0.00	926	0.00
la07	15 × 5	890	890	0.000	0.00	890	0.01
la08	15 × 5	863	863	0.000	0.00	863	0.00
la09	15 × 5	951	951	0.000	0.00	951	0.00
la10	15 × 5	958	958	0.000	0.00	958	0.00
la11	20 × 5	1222	1222	0.000	0.00	1222	0.00
la12	20 × 5	1039	1039	0.000	0.00	1039	0.00
la13	20 × 5	1150	1150	0.000	0.00	1150	0.00
la14	20 × 5	1292	1292	0.000	0.00	1292	0.00
la15	20 × 5	1207	1207	0.000	0.02	1207	0.15
la16	10 × 10	945	945	0.000	0.13	945	0.69
la17	10 × 10	784	784	0.000	0.11	784	0.13
la18	10 × 10	848	848	0.000	0.12	848	0.16
la19	10 × 10	842	842	0.000	0.16	842	0.51
la20	10 × 10	902	902	0.000	0.14	902	0.20
la21	15 × 10	1046	1046	0.000	2.68	1046.8	14.58
la22	15 × 10	927	927	0.000	0.72	927	5.50
la23	15 × 10	1032	1032	0.000	0.23	1032	0.24
la24	15 × 10	935	935	0.000	4.04	935.2	22.75
la25	15 × 10	977	977	0.000	1.51	977.2	10.97
la26	20 × 10	1218	1218	0.000	0.41	1218	0.44
la27	20 × 10	1235	1235	0.000	2.06	1235	27.79
la28	20 × 10	1211	1216	0.413	0.45	1216	0.79
la29	20 × 10	1152	1163	0.955	25.80	1164.3	32.50
la30	20 × 10	1355	1355	0.000	0.38	1355	0.40
la31	30 × 10	1784	1784	0.000	0.68	1784	0.70
la32	30 × 10	1850	1850	0.000	0.02	1850	0.04
la33	30 × 10	1719	1719	0.000	0.23	1719	0.70
la34	30 × 10	1721	1721	0.000	0.66	1721	0.67
la35	30 × 10	1888	1888	0.000	0.74	1888	0.77
la36	15 × 15	1268	1268	0.000	5.28	1268.4	16.01
la37	15 × 15	1397	1397	0.000	1.12	1397	3.89
la38	15 × 15	1196	1196	0.000	1.75	1199.2	14.18
la39	15 × 15	1233	1233	0.000	6.54	1235.4	21.19
la40	15 × 15	1222	1225	0.245	25.12	1227.1	17.95

## Concluding remarks

Towards generating both fast and high-quality solutions to the JSP, the SLENP has synergistically combined a construction method, a local search, and a large-neighborhood technique as a post-optimization component. Its construction technique constructs feasible schedules iteratively, one element at a time. The innovative feature of this component is the use of a forward-backward mechanism in scheduling activities.

The second component has been a local search, which starts with the initial schedules generated by the construction method. Consistent with all current effective neighborhood structures for the JSP, the employed local search has been founded on the concept of the critical block, which guides the construction neighbors. The innovative feature of this component is the use of two different neighborhoods that a parameter decides which to work in each round.

The third major component of the SLENP is the post-optimization method whose development has been inspired by the combination of the forward-backward shifting bottleneck procedure and the biased randomizing search. The reason for selecting this procedure as the post-optimization component for the SLENP has been twofold.

First, the shifting bottleneck heuristic is one of the most effective heuristics for the JSP which by sequencing the bottleneck machine successively, can provide high quality solutions for the JSP. Second, equipping this mechanism with a forward-backward process can further improve the accuracy of this highly effective mechanism.

By using these components, the SLENP can find solutions with high quality in a matter of seconds. This indicates that the components of the procedure act synergistically. Towards its enhancement, three major directions can be envisaged for sketching the procedure.

First, since a large portion of execution time is spent on calculating the values of the makespan, a faster evaluation technique, which without explicit calculation can estimate the makespan, can lead to producing solutions with higher quality through increasing search efficiency.

Second, in a parallel environment, various local searches can simultaneously operate to cooperatively locally optimize various parts of the same encoding. In this parallel environment, the local searches can communicate with one another so that each local search can ignore those parts of the encoding fixed by other local searches and concentrate only on manipulating its own part.

Third, since in the employed tabu search, the management of tabu list, which, as a short term memory, keeps the forbidden moves, has played a critical role in the quality of the overall result, other possible mechanisms in managing the tabu list can be tested. One promising mechanism is an adaptive tabu list, which based on a feedback received from the corresponding fitness landscape, can alternate between a fixed list and a random-sized list. Such a flexible tabu list, whose size is determined adaptively, can properly adjust the short term memory of the search and can possibly lead to higher quality solutions.
